# Measuring adolescents' HRQoL via self reports and parent proxy reports: an evaluation of the psychometric properties of both versions of the KINDL-R instrument

**DOI:** 10.1186/1477-7525-7-77

**Published:** 2009-08-26

**Authors:** Michael Erhart, Ute Ellert, Bärbel-Maria Kurth, Ulrike Ravens-Sieberer

**Affiliations:** 1 Child Public Health, Department of Psychosomatics in Children and Adolescents, University Medical Center Hamburg-Eppendorf, Martinistr. 52, D-20246 Hamburg, Germany; 2 Department of Health Reporting, Robert Koch-Institut, Seestr. 10, D-13353 Berlin, Germany

## Abstract

**Background:**

Several instruments are available to assess children's health-related quality of life (HRQoL) based on self reports as well as proxy reports from parents. Previous studies have found only low-to-moderate agreement between self and proxy reports, but few studies have explicitly compared the psychometric qualities of both. This study compares the reliability, factorial validity and convergent and known group validity of the self-report and parent-report versions of the HRQoL KINDL-R questionnaire for children and adolescents.

**Methods:**

Within the nationally representative cross-sectional German Health Interview and Examination Survey for Children and Adolescents (KiGGS), 6,813 children and adolescents aged 11 to 17 years completed the KINDL-R generic HRQoL instrument while their parents answered the KINDL proxy version (both in paper-and-pencil versions). Cronbach's alpha and confirmatory factor-analysis models (linear structural equation model) were obtained. Convergent and discriminant validity were assessed by calculating the Pearson's correlation coefficient for the Strengths and Difficulties Questionnaire. Known-groups differences were examined (ANOVA) for obese children and children with a lower familial socio-economic status.

**Results:**

The parent reports achieved slightly higher Cronbach's alpha values for the total score (0.86 vs. 0.83) and most sub-scores. Confirmatory factor analysis revealed an acceptable fit of the six-dimensional measurement model of the KINDL for the parent (RMSEA = 0.07) and child reports (RMSEA = 0.06). Factorial invariance across the two versions did not hold with regards to the pattern of loadings, the item errors and the covariation between latent concepts. However the magnitude of the differences was rather small. The parent report version achieved slightly higher convergent validity (r = 0.44 – 0.63 vs. r = 0.33 – 0.59) in the Strengths and Difficulties Questionnaire. No clear differences were observed for known-groups validity.

**Conclusion:**

Our study showed that parent proxy reports and child self reports on the child's HRQoL slightly differ with regards to how the perceptions, evaluations and possibly the affective resonance of each group are structured and internally consistent. Overall, the parent reports achieved slightly higher reliability and thus are favoured for the examination of small samples. No version was universally superior with regards to the validity of the measurements. Whenever possible, children's HRQoL should be measured via both sources of information.

## Background

Self-report questionnaires are regarded as the primary method for assessing health-related quality of life (HRQoL) in adults [1] as well as in children once they have reached a certain age and level of cognitive development [2]. However, there are also numerous proxy report measures available to assess the HRQoL of children and adolescents.

Several reviews and studies have examined the agreement between mental health and well-being reports made by parents and those made by the children themselves [3,4]. Studies involving healthy children found that parents generally proxy report higher mental health and well-being than the children do, whereas parents of children with chronic conditions tend to report lower QoLs than the children themselves. No consistent findings have been reported regarding the influence of other potential determinants of parent-child concordance, such as the child's age or gender or socio-economic variables. [4,5]. The level of agreement between proxy reports and children's self reports has also been found to vary between different aspects of HRQoL [3,4,6].

These results suggest that proxy ratings should be considered carefully as a potential substitute for self-report ratings [7]; it has been argued that proxy reports could also be regarded as providing complementary information about youths' mental health and well-being [3,6]. Different authors have emphasised that self reports and proxy reports constitute important complementary sources of information on children's QoLs. [3,6,8]. According to these authors, discrepancies between self reports and proxy reports might not be an indication of inaccuracy or bias in either data source. Instead, these differences could be regarded as validly reflecting each respondent's perspective [9]. At a minimum, the usefulness of proxy measures has been shown when assessing the mental health and well-being of children who are too immature or who have cognitive deficiencies [10,11].

However, to better judge the usefulness of the two sources of information, it is also important to study and compare the psychometric properties of self-report and parent-report measures and indicators. Proxy reports provide at least a partial view of a child's mental health and well-being [9] possibly complemented by important additional information from the parents. Thus, from a theoretical point of view, some differences in the validity of certain HRQoL determinants could be expected. Several scientific papers have described the psychometric properties of child and adolescent self-report instruments. Similarly, the psychometric properties of the corresponding parent-report versions have been examined (see [2,3,12] for an overview). Yet, few studies have explicitly focused on comparing the test-theoretical properties of the self-report and parent-report versions [13], for example by testing for statistically significant differences in Cronbach's alpha coefficients or validity coefficients, even though this is an important question to study. In epidemiological studies, low reliability and validity of HRQoL measures could lead to underestimating the impact of certain risk factors on the HRQoL of children and adolescents, which in turn could lead to overlooking significant health care and prevention needs.

Steele [14] found a different factor structure between the child self-report and the parent proxy-report versions of an oral health quality of life measure. A study by le Coq [13] found less random variance in the parent reports and higher score differences between groups with *a priori *expected differences in QoL when compared to the children's self reports. The parent-report scores also displayed larger (but not statistically significant) sensitivity for changes than did the children's self reports. Most studies reporting the psychometric properties of self-report and parent proxy-report versions observed similar internal consistencies for item responses [15-20]. However, for a paediatric psychiatric population [21] and a population of children with Asthma [22], higher Cronbach's alpha values were reported for parent-reported HRQoL scales compared to the children's self reports.

This paper sets out to examine the psychometric properties of the child self-report and the parent proxy-report versions of the KINDL-R Quality of Life measure [23], one of today's widely used generic HRQoL measures for children and adolescents. This study explicitly tested which version provides better psychometric properties by using inferential tests and *a priori*-specified criteria for meaningful differences in these psychometric properties.

The first psychometric property of interest is the dimensionality of the assessment. Analyses of this property could reveal whether the children themselves and their parents perceive and judge the children's health and life situations along similar dimensions, rather than operating within differentially structured perception and evaluation frames. This information is important to know because it is related to the validity of the measurement. Second, it is important to test whether the items within a particular HRQoL dimension are answered in an internally consistent manner, which is important for the reliability of the measurement. Third, it is important to assess whether the self ratings and parent ratings display similar patterns of association with aspects of theoretical relevance for HRQoL. This analysis refers to the convergent and discriminant validity of the two versions. Lastly, it is important to determine how well self and parent reports can discriminate between groups with *a priori *expected differences in HRQoL (known-groups validity).

This paper does not examine the self-proxy agreement/disagreement itself in depth, as this topic will be discussed in another paper (Ellert et al.: Agreement between self-rated and parent rated HRQoL in the KINDL-R. Results from the national representative German Health examination and Interview survey for Children and Adolescents (KiGGS), submitted).

## Methods

### Design, sample and procedure

This study was part of the German Health Interview and Examination Survey for Children and adolescents (KiGGS). The KiGGS study is a cross-sectional nationally representative general population and community-based survey in which a total of 17,641 children and adolescents aged 0 to 17 years were examined. The participants were medically and physically examined and tested. Parents filled in an extensive self-administered questionnaire including psychological and psychosocial instruments; children and adolescents older than 11 years also filled in a questionnaire themselves. The data were collected from May 2003 to May 2006 in 167 representatively selected sample points all over Germany. The objectives, procedures, design and measurements of the KiGGS are described in detail elsewhere [24]. The study was approved by the Charité-Universitätsmedizin Berlin ethics committee and the Federal Office for the Protection of Data.

The overall response rate was 66.6%. The current analyses were based on the health data of 6,813 children and adolescents aged 11 to 17 years. The statistical analyses were restricted to cases in which both the children's and the parents' responses on the KINDL were available.

### Measures

The HRQoL of children and adolescents was assessed using the generic KINDL-R questionnaire [23]. The KINDL-R questionnaire consists of 24 items covering six dimensions (referring to the past week): Physical well-being (e.g., felt sick), Emotional well-being (e.g., felt fearful or insecure), Self-worth (e.g., was happy with myself), Well-being in the family (e.g., felt comfortable at home), Well-being related to friends/peers (e.g., got along with friends), and School-related well-being (e.g., was afraid of getting bad grades). Each item provides five answer categories: never, seldom, sometimes, often and always. Item responses were coded with values between 1 and 5, with higher values indicating "better" HRQoL ratings. A total HRQoL score was calculated for all 24 items. The item scores per dimension (and the total score) were added and transformed into values between 0 and 100. The KINDL-R questionnaire includes a child and adolescent self-assessment version and an external-assessment version (to be completed by the parents).

The Strength and Difficulties Questionnaire (SDQ) [25] was applied as a brief behavioural screening questionnaire for children and teenagers to survey mental health symptoms and positive attitudes. Both the adolescent self-report version and the parent proxy-report version were applied. Both versions assess positive or negative attributes using 25 items focusing on five dimensions: Emotional symptoms (e.g., often unhappy, sad or tearful), Conduct problems (e.g., very angry and often lose temper), Hyperactivity/inattention (e.g., constantly fidgeting or squirming), Peer relationship problems (e.g., get on better with adults than with people of own age) and Prosocial behaviour (e.g., helpful if someone is hurt, upset or feeling ill). Each item is scored on a 3-point scale with 0 = not true, 1 = somewhat true, and 2 = certainly true; higher scores indicate greater problems except for in the Prosocial behaviour dimension, for which a higher score indicates more positive behaviour. Item scores are summed into subscores ranging from 0–10. Subscores for the four problem areas are summed up to generate a total difficulties score (0–40).

The children's weight and height were assessed by the interviewers using a standardised procedure. According to the conventions established by Cole et al. [26], the children's body mass indices were classified as extreme underweight, underweight, normal weight, overweight or obese.

Socio-economic status was determined using the 'Winkler Index' [27], which takes into account income, education and occupation (parental reports) and classifies households by low, middle or high socio-economic status.

Children's special health care needs, as an expression of chronic illness, were assessed with the Children with Special Health Care Needs (CSHCN) Screener [28]. The CSHCN comprises an array of five questions that are to be answered by the parents. These questions refer to (A) prescription medicine, (B) medical, psychosocial or pedagogical support, (C) functional limitations, (D) special therapies (physiotherapy, ergotherapy or speech therapy) and (E) treatments and consultations associated with emotional, developmental or behavioural problems. Children are classified depending on whether they need or do not need special health-related services.

### Statistical Analyses

The statistical analyses were based on weighted sample data to represent the age, gender, regional and citizenship structure of the German population (reference data 31.12.2004). The number of cases reported in the tables and in the text refers to weighted data and thus might deviate from the number of cases reported in the former description of the sample.

Basic psychometric item characteristics were calculated for each KINDL version: mean item score, SD and the corrected item-total correlation. To assess the reliability of the measurement, the Cronbach's alpha coefficient was computed. Corrected item-total correlations of 0.30 and more as well as Cronbach's alpha values above 0.70 were considered acceptable [29]. Cronbach's alpha values were compared across the two KINDL versions using Feld Tests for statistical significant differences [30].

The validity of the KINDL six-dimensional measurement model was tested by means of a linear structural equation model [31]. A confirmatory factor analysis was conducted using the LISREL 8 software. The identifiability of the model parameters was ensured by loading each observed variable on only one latent construct and by fixing the variance of each latent variable to one. The subsequent complete standardisation of the model enabled correct parameter estimates [32]. The database for the unweighted least squares (ULS) estimation of the model parameters was the polychoric correlation matrix of the observed indicators. As the ULS estimation procedure does not require multivariate normal distribution of the data, no *a priori *normalisation of the observed variables was applied [33]. The goodness of fit of the model was assessed by the Root Mean Square Residual (RMSEA). A RMSEA less than 0.6 (0.8) was taken as an indicator of excellent (adequate) fit between the specified model and the data [34]. The Comparative Fit Index (CFI) and the Adjusted Goodness-of-Fit Index (AGFI) were also reported. Loadings of 0.4 that furthermore exceeded any cross-loading were taken as indicators of sufficient representation of the common factor through the item.

To test for factorial invariance across the self- and proxy-report versions, a hierarchical sequence of multi-wave confirmatory factor analysis models was implemented, with the "multi-waves" defined by the test data from the KINDL self report and the parent proxy report respectively: first, all model parameters were estimated separately for each mode of administration (waves). Next, the factor loading estimates were forced to be equal across both modes. The next model imposed similar item-error variances across the different modes. The final, most restricted model furthermore forced the correlation between the six latent dimensions to be equal across the self-and parent-report versions. The likelihood ratio test was used to assess whether the more restricted model resulted in a statistically significant worse goodness of fit.

The level of agreement between self and proxy ratings was assessed with the intra-class correlation coefficient (two-way mixed effects, absolute agreement).

The pattern of Pearson's correlation between the KINDL scales and the SDQ parent- and self-report scales was calculated for each KINDL version. The KINDL dimensions were examined to assess whether they displayed at least moderate correlation (r > 0.3) with SDQ scales addressing emotional or behavioural aspects that are considered as determinants for the particular HRQoL domain. These correlations should be higher than correlations with aspects considered less relevant for the particular domain. Moderate correlations were expected. Although the SDQ addresses constructs different from those in the KINDL, we considered these analyses as tests for convergent and discriminant validity.

We tested which KINDL version (self or proxy) displayed stronger convergent validity. The Pearson's correlation coefficients for the two versions were transformed into Fisher's Z-values and the differences were computed. Differences of 0.1 – 0.29 in the Fisher's Z-values were classified as small effect sizes; differences of 0.3 – 0.49 were classified as medium effect sizes and those above 0.5 as large [35].

To test for known-groups validity, we used ANOVA to assess whether children with special health care needs, obese children and children with a lower familial socio-economic status display lower HRQoL in the KINDL scores (three separate analyses). Due to the generic nature of the KINDL-R effect, only small effect sizes were expected for differences in socio-economic status and weight status. For children with and without special health care needs, a medium-to-large effect size was expected. To test for statistically significant differences in known-groups validity between the two KINDL versions, the statistical interaction between the KINDL versions and the grouping was specified and tested.

The actual sample size of n = 7,166 respondents (including parent and self reports) allowed the detection of differences between correlation coefficients (corrected item-total correlation; correlation between KINDL and SDQ scales) of a magnitude of delta-r = 0.1 (small effect [35]) with a statistical power of p = 0.99 (two-tailed alpha < 0.05). In the ANOVA, the actual sample size also allowed the detection of a small interaction effect (f-effect size = 0.1 [35]) between modes of administration and an HRQoL-relevant grouping with a statistical power of p = 0.99 (two-tailed alpha < 0.05).

The statistical analyses were conducted with SPSS 15, Lisrel 8.7 and MS-Excel (Feldt Test) and were repeated across age-groups (11 – 13 versus 14 – 17 years).

## Results

### Sample characteristics

Table 1 shows the data that were available from 3,017 children aged 11–13 years and 4,598 adolescents aged 14–17 years. About 48.7% were female and 16.1% had an immigration background with at least both parents born outside the country [36]. About 17.5% were classified as having special health care needs as an indicator of a chronic health condition. Proxy report data were available for 7,166 cases. In 82.8% of cases, the proxy was the mother and in 11.4% it was the father. The mean age of mothers was 41.9 years and the mean age of fathers was 44.9 years. The real household income ranged from < 1500 Euros (17.8%) to > 3000 Euros (30.0%), with 25.4% reporting an income between 1500 and 2250 Euros and 26.9% reporting an income between 2250 and 3000 Euros. According to the Winkler Index, 25.3% of the families could be classified as having a low socio-economic status, 47.2% as having a medium socio-economic status and 27.4 as having a high socio-economic status.

**Table 1:** Sample Characteristics

	Weighted cases
Children (n = 7649)	
Mean age years (SD)	14.12 (1.98)
11–13 years (%)	39.6
14–17 years (%)	60.4
girls (%)	48.7
Migration status yes	16.1
Special health Care Needs %	17.5

Parents (n = 7559)	
mother (%)	82.8
father (%)	11.4
both (%)	4.8
other (%)	1.0
Mean age mothers (SD)	41.85 (5.10)
Mean age fathers (SD)	44.87 (6.13)
Real household income	
<1500 (%)	17.8
1500–2250 (%)	25.4
2250–3000 (%)	26.9
>3000 (%)	30.0
Socioeconomic status	
High (%)	27.4
medium (%)	47.2
low (%)	25.3

Migration status: At least one parent born outside country; main speech at home not German.

Special health care needs: CSHCN Screener [28]

Real household income after taxes etc.

Socioeconomic Status: Winkler Index [27]

### Basic psychometric properties and internal consistency of item responses

Table 2 shows differences in the mean KINDL scores between self and proxy ratings. For the dimensions of Self-Esteem and School-related well-being, less random variation was observed in the parent reports, which also exhibited smaller confidence intervals for the means. Table 2 also reports the mean item scores and SDs of the KINDL items for both versions. Overall, the mean item scores were slightly higher for the parent reports while the SDs were slightly lower. For the self-report version, the corrected item-total correlation ranged from 0.28 to 0.50 for the total (parent reports: 0.27 to 0.63) and from 0.30 to 0.59 for the dimensions of Physical well-being, Psychological well-being, Self-esteem, and Family well-being (parent reports: 0.34 to 0.63). For the self-report dimensions of Friend- and School-related well-being, the corrected item-total correlations ranged from 0.22 to 0.43 and from 0.17 to 0.40, respectively (parent reports: 0.24 to 0.59 and 0.34 to 0.45). On average, the Cronbach's alpha values were lower for the self-report version and ranged from 0.53 to 0.72 for the sub-dimensions. For the total score, a Cronbach's alpha of 0.83 was obtained. For the parent-report version, the Cronbach's alpha values ranged from 0.62 to 0.74 for the sub-dimensions. For the total score of the parent-report version, the Cronbach's alpha was 0.86. For both the self-report and the parent-report versions, slightly lower Cronbach's alpha values were observed in younger respondents aged 11 – 13 years compared to those 14 – 17 years old.

**Table 2:** Range of mean item score and standard deviation, Internal consistency of item responses.

	Mean	SD	Range of mean item score	Range of SD item score	Range of r_item-total_^a^	Cronbach alpha (11–13 years/14–17 years)
Self Report						
Total	72.58	10.35	3.03–4.63	0.65–1.25	0.28–0.50	0.82* (0.80*/0.83*)
Physical	70.61	16.50	3.41–4.30	0.87–1.05	0.30–0.44	0.59* (0.55*/0.61*)
Psychological	81.10	13.09	3.79–4.64	0.68–0.83	0.31–0.49	0.59* (0.52*/0.62*)
Self-Esteem	58.29	18.42	3.03–3.55	0.90–1.21	0.37–0.55	0.68 (0.68/0.69)
Family	81.93	15.71	4.08–4.44	0.80–0.96	0.41–0.59	0.72* (0.63*/0.76)
Friends	77.43	14.99	3.85–4.56	0.65–1.14	0.22–0.43	0.53* (0.53*/0.53*)
School	66.14	17.22	3.37–4.05	0.78–1.25	0.17–0.40	0.53* (0.51*/0.53*)

Parent Report						
Total	74.23	10.30	3.44–4.55	0.65–1.04	0.27–0.63	0.86 (0.86/0.86)
Physical	74.11	17.35	3.60–4.28	0.88–1.01	0.34–0.59	0.70 (0.67/0.72)
Psychological	79.19	13.20	3.83–4.55	0.67–.85	0.38–0.53	0.66 (0.64/0.67)
Self-Esteem	67.29	15.19	3.44–3.92	0.76–1.00	0.41–0.54	0.68^ns ^(0.66*/0.68^ns^)
Family	76.38	15.09	3.57–4.33	0.71–.95	0.47–0.63	0.74 (0.74/0.74*)
Friends	77.07	14.15	3.70–4.37	0.66–.91	0.24–0.59	0.64 (0.65/0.64)
School	71.41	15.85	3.56–4.17	0.77–1.05	0.34–0.45	0.62 (0.59/0.62)

Corrected item total correlation and Cronbach alpha coefficient: Range within scales

Standard Errors (SE) for the means = self report: 0.12 – 0.21 vs. parent report: 0.12 – 0.20 (95% confidence intervals = Mean +/- 1.96*SE)

^a ^Corrected for overlap.

* statistically significant smaller Cronbach alpha between self and parent proxy report (p < 0.01) in Feldt Test [30]

### Confirmatory factor analysis

A two-wave confirmatory factor analysis model [31] was specified according to the six-dimensional KINDL measurement model. The two waves represented the self-report and the parent-report versions. A series of hierarchical linear structural equation models with different degrees of equalisation of parameters between the two waves (self/parent version) were implemented. The first model, with separate estimation of parameters for each version, resulted in an acceptable goodness of fit based on the RMSEA = 0.066. Separate goodness-of-fit evaluations for the self-report and the parent-report versions showed similar results (self report: RMSEA = 0.064, AGFI = 0.944; parent report: RMSEA = 0.069, AGFI = 0.965). The estimated factor loadings ranged from 0.45 to 0.83 for the self-report version and from 0.47 to 0.85 for the parent-report version (Table 3). None of the item cross loadings exceeded the item loadings on the intended latent construct for either the self-report or the parent-report version. The factor loadings were transformed into Fisher's Z values and the differences across versions were calculated. The differences in Fisher's Z values ranged from 0.01 (marginal effect) to 0.32 (moderate effect). The median difference was 0.14, indicating a small effect.

**Table 3:** Confirmatory factor analysis – separate estimation of factor loadings

	Self Report (K)						Parent Report (P)					
Dimension	K1	K2	K3	K4	K5	K6	P1	P2	P3	P4	P5	P6
Items												
Felt ill	**0.53**	0.36	0.23	0.24	0.24	0.30	**0.69**	0.45	0.35	0.24	0.24	0.32
In pain	**0.48**	0.33	0.21	0.22	0.22	0.27	**0.55**	0.36	0.28	0.20	0.20	0.26
Tired	**0.62**	0.42	0.27	0.28	0.28	0.35	**0.74**	0.49	0.38	0.26	0.26	0.34
Energy	**0.59**	0.40	0.26	0.27	0.27	0.33	**0.68**	0.44	0.34	0.24	0.24	0.31

Fun	0.38	**0.55**	0.28	0.31	0.45	0.33	0.43	**0.66**	0.52	0.45	0.49	0.39
Bored	0.31	**0.46**	0.24	0.26	0.37	0.27	0.39	**0.60**	0.47	0.41	0.44	0.35
Alone	0.50	**0.73**	0.38	0.42	0.60	0.44	0.45	**0.69**	0.54	0.47	0.51	0.40
Scared	0.41	**0.59**	0.31	0.34	0.48	0.35	0.41	**0.63**	0.49	0.43	0.46	0.37

Proud	0.28	0.34	**0.65**	0.23	0.31	0.29	0.31	0.48	**0.61**	0.38	0.39	0.38
On Top	0.33	0.39	**0.76**	0.27	0.36	0.33	0.43	0.66	**0.85**	0.53	0.55	0.53
Pleased	0.25	0.30	**0.58**	0.21	0.27	0.26	0.30	0.47	**0.60**	0.37	0.38	0.37
Ideas	0.22	0.26	**0.50**	0.18	0.24	0.22	0.28	0.43	**0.55**	0.35	0.35	0.34

Parents	0.36	0.44	0.28	**0.78**	0.34	0.45	0.30	0.57	0.52	**0.83**	0.40	0.43
Home	0.38	0.47	0.30	**0.83**	0.37	0.48	0.31	0.60	0.54	**0.87**	0.42	0.45
Quarrels	0.30	0.37	0.23	**0.65**	0.29	0.38	0.22	0.43	0.39	**0.62**	0.30	0.32
Restricted	0.22	0.28	0.17	**0.49**	0.21	0.28	0.20	0.38	0.35	**0.56**	0.27	0.29

Friends	0.21	0.37	0.21	0.20	**0.45**	0.22	0.17	0.36	0.31	0.24	**0.49**	0.17
Success	0.29	0.51	0.29	0.28	**0.63**	0.31	0.27	0.56	0.49	0.37	**0.76**	0.27
Got along	0.30	0.54	0.31	0.29	**0.66**	0.32	0.28	0.57	0.50	0.38	**0.78**	0.28
Different	0.24	0.42	0.24	0.23	**0.52**	0.25	0.22	0.45	0.39	0.30	**0.61**	0.22

School-work	0.35	0.38	0.28	0.37	0.31	**0.63**	0.34	0.43	0.46	0.38	0.26	**0.74**
Interesting	0.25	0.27	0.20	0.26	0.22	**0.45**	0.31	0.40	0.42	0.34	0.24	**0.67**
Future	0.29	0.31	0.23	0.30	0.25	**0.52**	0.22	0.27	0.29	0.24	0.17	**0.47**
Bad marks	0.27	0.29	0.21	0.28	0.23	**0.48**	0.23	0.29	0.30	0.25	0.17	**0.49**

Complete standardized parameter estimation

Goodness of fit self report: RMSE = 0.064; CFI = 0.931; AGFI = 0.944.

Goodness of fit parent report: RMSE = 0.069; CFI = 0.952; AGFI = 0.965.

For the self-report version, the correlation between the latent dimensions ranged from 0.36 to 0.82. The latent dimensions of the parent-report version had correlations ranging from 0.36 to 0.78. The largest differences between the self- and proxy-report versions were found for the correlation between the dimensions of Self-esteem and Family well-being, as well as for the correlation between the dimensions of Self-Esteem and Psychological well-being. Table 4 shows that, for the self-report version, these correlations were 0.36 and 0.52, respectively. For the parent-report version, these correlations were 0.63 and 0.78 respectively. The correlations were transformed into Fisher's Z values, and the differences were calculated across the two versions. The differences in the Fisher's Z-values ranged from 0.02 (marginal effect) to 0.47 (moderate to large effect). The median difference was 0.14, indicating a small effect.

**Table 4:** Confirmatory factor analysis – separate estimation of correlation between latent constructs (KINDL measurement dimensions)

Dimension	r	r	r	r	r	r
Self Report	K1	K2	K3	K4	K5	K6
K1 Physical						
K2 Psychological	0.69					
K3 Self-Esteem	0.44	0.52				
K4 Family	0.46	0.57	0.36			
K5 Friends	0.46	0.82	0.47	0.44		
K6 School	0.56	0.60	0.44	0.58	0.49	

Parent Report	P1	P2	P3	P4	P5	P6
P1 Physical						
P2 Psychological	0.65					
P3 Self-Esteem	0.51	0.78				
P4 Family	0.36	0.69	0.63			
P5 Friends	0.35	0.74	0.64	0.48		
P6 School	0.46	0.59	0.62	0.51	0.36	

Complete standardized parameter estimation

The goodness-of-fit results for the hierarchical series of confirmatory factor analyses are shown in Table 5. In the second model, the item loadings on the latent constructs were set to be equal for the self-report and the parent-report versions. This model achieved an RMSEA of 0.067. The difference in the likelihood ratio χ^2 ^values was statistically significant, indicating a better fit of the unrestricted model. The third model introduced equal error variances in the items. The RMSEA of this model was 0.069. The difference in the χ^2 ^values between models two and three was statistically significant: the more restricted model three achieved a statistically significant worse fit. The last model furthermore included an equal pattern of correlation between the latent variables (KINDL dimensions) for the self-report and the parent-report versions. This model again resulted in a statistically significant worse goodness of fit compared to the less restricted model three. The RMSEA was 0.070.

**Table 5:** Factorial Invariance between KINDL self and proxy version.

Model		RMSEA	CFI	AGFI	χ^2^	Df	p (Delta χ^2^)
1	Unrestricted	0.066	0.999	0.937	31532.14	1014	
2	Loadings	0.067	0.999	0.930	32560.19	1038	< 0.001
3	Error	0.069	0.999	0.921	35323.06	1062	< 0.001
4	Factors	0.070	0.999	0.919	36325.68	1077	< 0.001

Goodness of fit Indices issued from multi-wave factor analyses with different degrees of restriction

Model 1 = Unrestricted separate estimation of parameters; Model 2 = Loadings set to be equal; Model 3 = Error variances in items set to be equal; Model 4 = Factor correlation set to be equal.

The confirmatory factor analyses were repeated across age groups (11 – 13 years versus 14 – 17 years). The results showed no sizeable variation in the pattern of factor loadings and factor correlation across age groups for either the self reports or the parent reports (results not shown).

### Self-proxy agreement

Detailed information on the self-proxy agreement is reported in another publication. The intra-class correlation coefficient for the absolute agreement for the entire age range was 0.49 for the total score and ranged from 0.24 to 0.45 for the sub-dimensions.

### Convergent/discriminant and known-groups validity

To test for convergent and discriminant validity, the two KINDL versions were correlated with the SDQ self- and parent-report versions. It was expected *a priori *that the KINDL Psychological well-being dimension would display the highest correlation with the SDQ Emotional scale. The KINDL dimension of Family well-being was expected to show the highest correlation with the SDQ Conduct scale. For the KINDL dimension of Friend-related well-being, the highest correlation was expected with the SDQ Peer problems scale. The magnitude of these associations should at least be moderate. It was also expected that the total HRQoL would be most closely associated with general emotional and behavioural problems as measured by the SDQ Total difficulties score.

Table 6 shows that the KINDL self-report version displays the expected pattern of association with the SDQ self-report version. The KINDL dimensions of Psychological, Family-related and Friend-related well-being displayed convergent validity with coefficients between 0.33 and 0.49. The KINDL self-report total score showed the highest correlation with the SDQ self-report Total difficulties score (r = 0.57). Discriminant validity was indicated by the lower correlation of these KINDL dimensions with other SDQ scales. The KINDL self-report version also displayed convergent and discriminant validity with regard to the SDQ parent-report version, though the actual correlation coefficients were lower. However, the KINDL Psychological well-being dimension failed to achieve a convergent validity of r = 0.30 with the parent-rated SDQ Emotion scale. The actual correlation was r = 0.26.

**Table 6:** Association between KINDL-R and the Strengths and Difficulties Questionnaire

	KINDL Children's self-report						
	Total	Physical	Psychological	Self-esteem	Family	Friends	School
SDQ Self report							

Total	***-0.57***	-0.36	-0.41	-0.20	-0.37	-0.42	-0.48
Emotional	-0.47	-0.40	*-0.37*	-0.13	-0.25	-0.30	-0.38
Conduct	-0.35	-0.19	-0.22	-0.10	***-0.35***	-0.21	-0.32
Hyperactivity	-0.32	-0.17	-0.16	-0.17	-0.25	-0.14	-0.34
Peer Problems	-0.42	-0.20	-0.37	-0.14	-0.18	***-0.51***	-0.26
Prosocial^c^	0.25	0.06	0.16	0.18	0.19	0.18	0.19

SDQ Parent Report							

Total	***-0.33***	-0.17	-0.23	-0.18	-0.29	-0.19	-0.25
Emotional	-0.34	-0.24	*-0.26*	-0.19	-0.20	-0.20	-0.22
Conduct	-0.20	-0.07	-0.10	-0.10	***-0.32***	-0.05	-0.13
Hyperactivity	-0.16	-0.05	-0.07	-0.10	-0.19	0.00	-0.20
Peer Problems	-0.25	-0.12	-0.23	-0.11	-0.13	***-0.32***	-0.12
Prosocial^c^	0.16	0.02	0.11	0.11	0.23	0.07	0.09

	KINDL Children's self-report						
	Total	Physical	Psychological	Self esteem	Family	Friends	School

SDQ Self report							

Total	***-0.33***	-0.21	-0.24	-0.21	-0.21	-0.24	-0.26
Emotional	-0.26	-0.23	*-0.19*	-0.16	-0.10	-0.17	-0.20
Conduct	-0.19	-0.08	-0.14	-0.12	*-0.19*	-0.13	-0.14
Hyperactivity	-0.21	-0.11	-0.13	-0.15	-0.17	-0.06	-0.22
Peer Problems	-0.24	-0.14	-0.18	-0.13	-0.10	***-0.32***	-0.13
Prosocial^c^	0.13	0.01	0.12	0.12	0.09	0.09	0.11

SDQ Parent Report							

Total	***-0.63***	-0.34	-0.50	-0.43	-0.46	-0.43	-0.42
Emotional	-0.58	-0.38	***-0.48***	-0.34	-0.28	-0.33	-0.33
Conduct	-0.40	-0.12	-0.28	-0.24	***-0.44***	-0.22	-0.22
Hyperactivity	-0.37	-0.11	-0.23	-0.23	-0.28	-0.17	-0.26
Peer Problems	-0.42	-0.17	-0.35	-0.26	-0.20	***-0.53***	-0.24
Prosocial^c^	32	0.07	0.24	0.24	0.31	0.22	0.18

Pearson correlation coefficients

The KINDL parent-report version showed convergent validity with the parent-rated SDQ, with the actual correlation between dimensions with *a priori*-expected association ranging from 0.44 to 0.53. The total score on the parent-reported KINDL showed the highest correlation with the parent-reported SDQ Total difficulties score (r = 0.63). However, the KINDL parent version showed convergent and discriminant validity with the self-rated SDQ only in the KINDL Total score (r = 0.33 with SDQ Total difficulties score) and the Friend-related well-being dimension (r = 0.32 with SDQ Peer problems). Separate analyses for participants 11 – 13 years old and 14 – 17 years old showed a similar pattern of correlation between the KINDL and the SDQ across age groups (results not shown).

Regarding the known-groups analysis, we tested whether the KINDL could discriminate between children with and without special health care needs (CSHCN). Table 6 shows effect sizes of 0.04 to 0.27 (small effect size) for the mean difference in self-reported KINDL scores. For the parent-reported scores, effect sizes between 0.20 and 0.56 (medium effect size) were observed. Next, we examined which KINDL version better captured the *a priori*-expected differences between children with normal weight and those who were obese. Table 7 shows larger effect sizes for that difference in the parent-reported KINDL Total score and Physical well-being dimension (d = 0.31 and 0.26) than for the same dimensions in the KINDL self-report version (d = 0.25 and 0.18). Nevertheless, all these differences only represent small effects [36]. The KINDL self-report version displayed larger effect sizes for the impact of obesity on the dimensions of Self-esteem, Friends and School-related well-being. The actual d-effect sizes of 0.19, 0.28 and 0.23 represent small effects. For the corresponding parent-reported dimensions, only marginal effects were seen, as indicated by the d-effect sizes of 0.11, 0.08 and 0.11. Separate analyses for the 11- to 13-year olds and the 14- to 17-year olds showed remarkably different effect sizes for obesity in the KINDL self-report Total score (0.26 versus 0.07) and the Physical well-being (0.31 versus 0.11) and Self-esteem (0.05 versus 0.28) sub-dimensions as well as the KINDL parent-reported Physical well-being (0.58 versus 0.15) sub-dimension. Both KINDL versions showed that younger children are more affected by obesity than older children, except for in the Self-esteem dimension, in which older children were more affected.

**Table 7:** Impact of special health care need and obesity on HRQoL children self reports and parents proxy report

	Special Health Care Needs (CSHCN)				Weight status^a^				
	Yes	no	d-effect size		normal	over-weight	Obese	d-effect size^b^	
			all	(11–13/14–17 years)				all	11–13/14–17 years
N	1136	5917			5908	1227	438		
	Mean	Mean			Mean	Mean	Mean		
Self Report									
Total	70.73	73.20	-0.23**	-0.16/-0.29	72.92	71.81	70.31	0.25**	0.26/0.07
Physical	68.23	71.05	-0.27**	-0.08/-0.24	71.07	69.35	68.03	0.18**	0.31/0.11
Psychological	78.96	81.69	-0.21**	-0.21/-0.22	81.23	80.79	80.41	0.06 ns	0.13/0.02
Self-Esteem	56.75	58.76	-0.11**	0.01/-0.19	58.81	57.05	55.36	0.19**	0.05/0.28
Family	80.02	82.25	-0.14**	-0.11/-0.17	81.96	81.94	81.25	0.05 ns	0.08/0.02
Friends	74.46	78.07	-0.24**	-0.19/-0.29	77.77	77.07	73.62	0.28**	0.33/0.24
School	66.01	66.77	-0.04 ns	-0.08/-0.03	66.70	64.68	62.78	0.23**	0.15/0.27

Parent Report									
Total	69.53	75.22	-0.56**	-0.62/-0.52	74.68	73.18	71.48	0.31**	0.41/0.25
Physical	68.97	75.15	-0.36**	-0.36/-0.36	74.91	72.19	69.59	0.31**	0.58/0.15
Psychological	73.89	80.45	-0.50**	-0.54/-0.47	79.60	78.04	77.07	0.19**	0.22/0.06
Self-Esteem	62.81	68.34	-0.37**	-0.38/-0.37	67.71	66.30	64.87	0.09**	0.04/0.12
Family	72.15	77.13	-0.33**	-0.38/-0.30	76.61	75.59	75.51	0.07*	0.15/0.01
Friends	70.60	78.80	-0.55**	-0.63/-0.50	77.56	76.13	73.44	0.08**	0.17/0.02
School	68.79	71.99	-0.20**	-0.30/-0.16	71.76	70.89	67.86	0.05**	0.08/0.08

Standard errors (SE) for the means: normal weight = 0.13 – 0.24 (self) vs. 0.13 – 0.23 (parent); overweight = 0.29 – 0.53 (self) vs. 0.31 – 0.52 (parent); obese = 0.57 – 1.02 (self) vs. 0.52 – 0.86 (parent); 95% confidence intervals = mean +/- 1.96*SE)

^a ^Weight classification according to IOTF Cole et al. [26]

^b ^"d"-effect size for comparison between normal weight and obese (0.2 = small; 0.5 = medium; 0.8 = large effect)

* statistical significant (p < 0.05) F-value in ANOVA; ** statistical significant (p < 0.01) F-value in ANOVA

No statistically significant interaction was observed for mode * socio-economic status

All statistical interactions between mode (parent versus self) and CSHCN were statistically significant

All statistical interactions between mode (parent versus self) and weight status were statistically non-significant except for KINDL Psych (p = 0.011; "f" = 0.04 [marginal effect]).

The theoretical expected impact of a low socio-economic status (SES) on children and adolescents' HRQoL could be best detected with the parent-reported KINDL sub-dimension of School-related well-being and the parent-reported KINDL Total score. The d-effect sizes of 0.36 and 0.19 indicate small effects. The impact of low SES on HRQoL was remarkably different across age groups in the self-reported dimension of Self-esteem. While 11- to 13-year olds with low SES reported slightly higher self-esteem, the 14- to 17-year olds with low SES reported lower self-esteem than their peers with high SES (d-effect size = 0.17 versus -0.24). No such difference was seen in the parent reports. (Table 8).

**Table 8:** Impact of socio-economic status on HRQoL children self reports and parents proxy report

	Socio-economic status^a^				
	Low	Medium	High	d-effect size^b^	d-effect size^b^
				all	11–13/14–17 years
N	2036	3507	1880		
	Mean	Mean	Mean		
Self Report					
Total	71.89	72.71	73.38	-0.14**	-0.15/-0.11
Physical	70.13	70.61	71.35	-0.07 ns	-0.07/-0.09
Psychological	80.86	81.16	81.52	-0.05 ns	-0.17/0.01
Self-Esteem	57.55	58.40	58.92	-0.07*	0.17/-0.24
Family	81.79	81.72	82.59	-0.05 ns	-0.17/0.01
Friends	77.51	77.99	76.34	0.08**	0.02/0.10
School	63.56	66.31	69.65	-0.36**	-0.39/-0.37

Parent Report					
Total	73.44	74.23	75.10	-0.16**	-0.21/-0.13
Physical	72.49	74.22	75.64	-0.18**	-0.26/-0.14
Psychological	78.50	79.17	79.99	-0.11**	-0.14/-0.05
Self-Esteem	66.48	67.37	68.07	-0.07**	-0.08/-0.06
Family	76.28	76.17	76.84	0.04^ns^	-0.01/0.02
Friends	77.54	77.39	75.97	0.01^ns^	0.05/-0.01
School	69.24	71.16	74.13	-0.13**	-0.19/-0.12

Standard errors (SE) for the means: low SES = 0.24 – 0.45 (self) vs. 0.17 – 0.28 (parent); medium SES = 0.18 – 0.31 (self) vs. 0.17 – 0.30 (parent); high SES = 0.23 – 0.39 (self) vs. 0.23 – 0.39 (parent); 95% confidence intervals = mean +/- 1.96*SE)

^a^Socioeconomic status classification according to the Winkler Index [27].

^b^"d"-effect size for comparison between low and high socioeconomic status (0.2 = small; 0.5 = medium; 0.8 = large effect)

* statistical significant (p < .05) F-value in ANOVA; ** statistical significant (p < 0.01) F-value in ANOVA

No statistically significant interaction was observed for mode * socio-economic status

All statistical interactions between mode (parent versus self) and CSHCN were statistically significant

All statistical interactions between mode (parent versus self) and weight status were statistically non-significant except for KINDL Psych (p = 0.011; "f" = 0.04 [marginal effect]).

## Discussion

This study aimed to compare the internal consistency of item responses, factorial validity and invariance and the convergent and known-groups validity of the child-report version and the parent-report version of the KINDL-R [24], a generic HRQoL instrument for children and adolescents. In summary, the results indicated that both KINDL versions enable a reliable assessment of general HRQoL in children and adolescents. Both versions showed factorial validity with only slight invariance across the self-report and the parent-report versions. Both versions displayed convergent and discriminant validity and known-groups validity. Neither the parent-report version nor the self-report version was universally superior to the other.

Both KINDL versions enable reliable assessment of general HRQoL. The parents responded in only a slightly more consistent manner than the children. Similar results have been found in other studies [11,22]. These differences were slightly more pronounced in the younger age group (11 – 13 years old) than in the older age group (14 – 17 years old). Different factors might account for this finding: younger children might have a lower span of attention and concentration or more difficulties in recalling the aspects asked about in the survey [37]. On the other hand, though the KINDL claims to be valid for use in children from the age of 11 years on, some of the younger respondents might have difficulties in comprehending single words or expressions used in the item statements.

Both the KINDL self-report and the parent proxy-report versions displayed acceptable factorial validity: the theoretical six-dimensional measurement model of the KINDL fit the data well according to *a priori*-defined criteria and explained the correlation between the items well. Item loadings above 0.4 and low cross-loadings confirmed that the items are sufficient to represent the common factor in their respective measurement dimension.

Factorial invariance across the modes of administration could be not confirmed: there were statistically significant differences in the actual pattern and magnitude of item loadings, the item errors and the covariation between the latent measurement dimensions. However, the actual large sample size could lead to an overwhelming power to detect even small and practically meaningless differences. The magnitude of these differences could be classified as "moderate" only for some parameters. On average, the differences across the versions represent only small effects.

The examination of convergent validity overall showed that both the KINDL self-report and parent-report versions display convergent and discriminant validity [38] with regard to the pattern of association with emotional and behavioural problems. The KINDL **parent **report displayed better convergent and discriminant validity with **parent**-reported emotional and behavioural problems of children. The KINDL **self **report showed better convergent and discriminant validity with the **child**-reported emotional and behavioural problems. These results can be interpreted as evidence of convergent and discriminant validity, even considering that the SDQ addresses constructs different from those of the KINDL. However, we considered the SDQ scores of emotional or behavioural problems and strengths as determinants for particular HRQoL domains.

Both KINDL versions displayed known-groups validity. The parent report version showed higher validity coefficients – indicating a medium effect size – when discriminating between children with and without special health care needs. However, it is important to bear in mind that the special health care needs were assessed through parent ratings. The identical source of information might have increased the magnitude of the observed differences. The KINDL self-report version could better capture the theoretically expected impact of low socio-economic status, especially on school-related well-being. The parent proxy report, on the other hand, was more sensitive to the theoretical expected impact of obesity on children's HRQoL.

The effect sizes for these differences were only small in magnitude for SES and obesity and at most moderate for special health care needs. However, this result could be expected *a priori*: social determinants might reveal larger differences in small areas or local groups. Furthermore, the role of mediating and moderating factors such as community or ethnic belonging, social capital and personal coping abilities might play a major role. Such a complex analysis, however, was beyond the scope of our paper and is suggested for future analyses. The impact of obesity on HRQoL is best measured with disease-specific HRQoL modules. The KINDL offers such specific modules but its obesity module was not applied in the present study.

Additional limitations of this study relate to the examination of convergent and known-groups validity: there was little HRQoL-relevant information on health status and life situation available from third parties other than children and parents, such as clinical diagnoses or semi-structured clinical interviews. However, due to the so-called same source of information bias, the association between self-reported HRQoL and self-reported determinants as well as the relation between parent-reported HRQoL and parent-reported determinants is of limited value in determining which version exhibits better validity. Our results on convergent and discriminant validity as well as on known-groups validity thus capture only a limited sample of all relevant aspects of construct validity. Generalisation of the results is only possible for the aspects that were actually studied. What is also lacking is information on the stability of HRQoL scores over time as well as their sensitivity to change.

## Conclusion

Our study showed that parent proxy reports and children's self reports on the children's HRQoL differ with regards to how the perceptions, evaluations and possibly the affective resonance are structured and internally consistent. The advantages of the parent reports include their slightly greater internal consistency, which enables the accurate measurement of HRQoL even for small groups of children. For the examination of HRQoL of small groups of respondents, our results suggest a focus on the KINDL total score of the self-report version rather than the self-reported sub-dimensions. However, before carrying out such analyses, one should first clarify the aspects or determinants for which the HRQoL measurement should be sensitive. The decision on the source of information to be used should consider the particular aim and research question.

Additional research is needed to examine the cognitive processes and the affective correlates of the item-response behaviour of children and parents. This issue could be best studied in a qualitative examination. Further studies could also try to examine the stability of KINDL-R self reports and parent proxy reports over time and also the responsiveness over time. These areas of inquiry are important because measures that lack sensitivity to change might not be able to capture the effects of successful treatment and intervention for children [13]. Additional research is also needed in testing the convergent and discriminant as well as known-groups validity of the two KINDL versions, using additional information on health status and life situation.

## Competing interests

The authors declare that they have no competing interests.

## Authors' contributions

ME conducted the statistical analyses and wrote the manuscript. BK and URS were the principal investigators of the study; they designed the study's concept and supervised the writing of the manuscript. UE assisted in the statistical analyses and revised the manuscript. All authors have read and approved the final manuscript.

## References

[B1] Hays RD, Vickrey BG, Hermann BP, Perrine K, Cramer J, Meador K (1995). Agreement between self-reports and proxy reports of quality of life in epilepsy patients. Qual Life Res.

[B2] Ravens-Sieberer U, Erhart M, Wille N, Wetzel R, Nickel J, Bullinger M (2006). Generic health-related quality of life assessment in children and adolescents: methodological considerations. Pharmacoeconomics.

[B3] Eiser C, Morse R (2001). A review of measures of quality of life for children with chronic illness. Arch Dis Child.

[B4] White-Koning M, Arnaud C, Dickinson HO, Thyen U, Beckung E, Fauconnier J, McManus V, Michelsen SI, Parkes J, Parkinson K, Schirripa G, Colver A (2007). Determinants of Child-Parent Agreement in Quality-of-Life Reports: A European Study of Children With Cerebral Palsy. Pediatrics.

[B5] Robitail S, Simeoni MC, Ravens-Sieberer U, Bruil J, Auquier P (2007). Children proxies' quality-of-life agreement depended on the country using the European KIDSCREEN-52 questionnaire. J Clin Epidem.

[B6] Eiser C, Morse R (2001). Quality-of-life measures in chronic diseases of childhood. Health Technol Assess.

[B7] Jokovic A, Locker D, Guyatt G (2004). How well do parents know their children? Implications for proxy reporting of child health related quality of life. Qual Life Res.

[B8] De Civita M, Regier D, Alamgir AH, Anis AH, Fitzgerald MJ, Marra CA (2005). Evaluating health-related quality-of-life studies in paediatric populations: some conceptual, methodological and developmental considerations and recent applications. Pharmacoeconomics.

[B9] White-Koning M, Arnaud C, Bourdet-Loubère S, Bazex H, Colver A (2005). Subjective quality of life in children with intellectual impairment – how can it be assessed?. Developmental Medicine & Child Neurology.

[B10] Epstein AM, Hall JA, Tognetti J, Son LH, Conant L (1989). Using proxies to evaluate quality of life: can they provide valid information about patients' health status and satisfaction with medical care?. Med Care.

[B11] Lawford J, Volavka N, Eiser C (2001). A generic measure of quality of life for children aged 3–8 years: results of two preliminary studies. Pediatr Rehabil.

[B12] Solans M, Pane S, Estrada M-D, Serra-Sutton V, Berra S, Herdman M, Alonso J, Rajmil L (2008). Health-related quality of life measurement in children and adolescents: A systematic review of generic and disease specific instruments. Value in Health.

[B13] Le Coq EM, Boeke AJP, Bezemer PD, Colland VT, van Eijk JTM (2000). Which source should we use to measure quality of life in children with asthma: The children themselves or their parents?. Qual Life Res.

[B14] Steele MM (2008). Reliability and validity of the Oral Health Scale of the PedsQL: Measuring the relationship between child oral health and health-related quality of life. Dissertation Abstracts International: Section B: the Science and Engineering.

[B15] Hainsworth KR, Davies WH, Khan KA, Weisman SJ (2007). Development and preliminary validation of the Child Activity Limitations Questionnaire: Flexible and efficient assessment of pain-related functional disability. Journal of Pain.

[B16] Laaksonen C, Aromaa M, Heinonen OJ, Suominen S, Salantera S (2007). Paediatric health-related quality of life instrument for primary school children: Cross-cultural validation. Journal of Advanced Nursing.

[B17] Upton P, Maddocks A, Eiser C, Earnest PM, Williams J (2005). Development of a measure of the health-related quality of life of children in public care. Child: Care, Health and Development.

[B18] Ravens-Sieberer U, the European KIDSCREEN Group (2006). The KIDSCREEN Questionnaires – Quality of life questionnaires for children and adolescents – handbook.

[B19] Verrips EGH, Vogels TGC, Koopman HM, Theunissen NCM, Kamphuis RP, Fekkes M, Wit JM, Verloove-Vanhorick SP (1999). Measuring health-related quality of life in a child population. European Journal of Public Health.

[B20] Varni JW, Burwinkle TM, Seid M (2006). The PedsQLTM 4.0 as a school population health measure: Feasibility, reliability, and validity. Qual Life Res.

[B21] Bastiaansen D, Koot HM, Bongers IL, Varni JW, Verhulst FC (2004). Measuring quality of life in children referred for psychiatric problems: Psychometric properties of the PedsQL-super(TM) 4.0 generic core scales. Qual Life Research.

[B22] Varni JW, Burwinkle TM, Rapoff MA, Kamps JL, Olson N (2004). The PedsQL-super(TM) in pediatric asthma: Reliability and validity of the Pediatric Quality of Life Inventory-super(TM) generic core scales and asthma module. J Beh Medicine.

[B23] Ravens-Sieberer U, Schumacher J, Klaiberg A, Brähler E (2003). Der Kindl-R Fragebogen zur Erfassung der gesundheitsbezogenen Lebensqualität bei Kindern und Jugendlichen – Revidierte Form. Diagnostische Verfahren zu Lebensqualität und Wohlbefinden.

[B24] Kurth B-M, Kamtsiuris P, Hoelling H, Schlaud M, Doelle R, Ellert U, Kahl H, Knopf H, Lange M, Mensinck GBM, Neuhauser H, Schaffrath Rosario A, Scheidt-Nave C, Schenk L, Schlack R, Stolzenberg H, Thamm M, Thierfelder W, Wolf U (2008). The challenge of comprehensively mapping children's health in a nation-wide health survey: Design of the German KiGGS-Study. BMC Public Health.

[B25] Goodman R (1997). The Strengths and Difficulties Questionnaire: A research note. Journal of Child Psychology, Psychiatry, and Allied Disciplines.

[B26] Cole TJ, Bellizzi MC, Flegal KM, Dietz WH (2000). Establishing a standard definition for child overweight and obesity worldwide: international survey. British Medical Journal.

[B27] Winkler J, Stolzenberg H (1999). Der Sozialschichtindex im Bundesgesundheitssurvey. Gesundheitswesen.

[B28] Bethell C, Read D, Stein RE, Blumberg SJ, Wells N, Newacheck PW (2002). Identifying children with spezial health care needs: developement and evaluation of a short screening instrument. Ambulatory Pediatrics.

[B29] Nunnally JC, Bernstein IR (1994). Psychometric Theory.

[B30] Feldt LS (1980). A test of the hypothesis that Cronbach's alpha reliability coefficient is the same for two tests administered to the same sample. Psychometrika.

[B31] Jöreskog KG, Sörbom D (2001). LISREL 8: Users's Reference Guide.

[B32] McDonald RP, Ho MHR (2002). Principals and Practice in Reporting Structural Equation Analyses. Psychological Methods.

[B33] Jöreskog KG, Sörbom D (2002). PRELIS 2: Users's Reference Guide.

[B34] Hu L, Bentler PM (1999). Cutoff Criteria for Fit Indexes in Covariance Structure Analysis. Conventional Criteria Versus New Alternatives. Structural Equation Modeling.

[B35] Cohen J (1988). Statistical Power Analysis for the Behavioural Sciences.

[B36] Schenk L, Ellert U, Neuhauser H (2007). Children and adolescents in Germany with a migration background. Methodological aspects in the German Health Interview and Examination Survey for Children and Adolescents (KiGGS). Bundesgessundheitsbl – Gesundheitsforsch – Gesundheitsschutz.

[B37] Ungar WJ, Mirabelli C, Cousins M, Boydell KM (2006). A qualitative analysis of a dyad approach to health-related quality of life measurement in children with asthma. Soc Sci Med.

[B38] Campbell DT, Fiske DW (1959). Convergent and discriminant validation by the multitrait-Multimethod Matrix. Psychological Bulletin.

